# ANTERIOR INTEROSSEOUS NERVE TRANSFERS FOR THE TREATMENT OF RADIAL NERVE INJURIES

**DOI:** 10.1590/1413-785220253303e277311

**Published:** 2025-08-18

**Authors:** Edie Benedito Caetano, Luiz Angelo Vieira, Vinicius Santos Bueno, Túlio Stefanin Volpiani, Victor Hugo Monfrin Torres, Andrea Elisa Donovan Giraldo

**Affiliations:** 1 Pontificia Universidade Catolica de Sao Paulo (PUC), Faculdade de Ciencias Medicas e da Saude, Sorocaba, Sao Paulo, SP, Brazil.

**Keywords:** Cadaver, Dissection, Forearm, Muscles, Reconstructive Surgical Procedures, Cadáver, Dissecação, Antebraço, Músculos, Procedimentos Cirúrgicos Reconstrutivos

## Abstract

**Objectives:**

evaluate the anatomical characteristics and variations of the anterior interosseous nerve (AIN) and define the feasibility of transferring the pronator quadratus branch (PQB) to the posterior interosseous nerve (PIN) without tension.

**Materials and methods:**

Fifty upper limbs of 25 male adult cadavers were dissected, 20 were prepared and five were fresh cadavers.

**Results:**

The AIN originated from the median nerve in an average of 5.2 cm distal to the intercondylar line. In 29 limbs, it originated from the posterior fascicles of the median nerve, while in 21 specimens, from the posterolateral fascicles. In 2 specimens, two branches for the AIN were present. The PIN was studied in 30 limbs, we identified its origin in the radial nerve in all limbs. In 23 limbs, the branches to the supinator muscle originated from the PIN proximally to the Fröhse arcade, in 7 members distally.

**Conclusion:**

The PQB was reliably present in all dissected forearms and presented variations only in its diameter. The PQB could be transferred to PIN without tension in all specimens even with full range of motion of the forearm. ***Level of Evidence IV; Case Series.***

## RESUMO

**Objetivos:**

avaliar as características e variações anatômicas do nervo interósseo anterior (NIA) e definir a viabilidade da transferência do ramo pronador quadrado (RPQ) para o nervo interósseo posterior (NIP) sem tensão.

**Materiais e métodos:**

Cinquenta membros superiores de 25 cadáveres adultos do sexo masculino foram dissecados, 20 foram preparados e cinco eram cadáveres frescos.

**Resultados:**

O NIA originou-se do nervo mediano em média 5,2 cm distal à linha intercondilar. Em 29 membros originou-se dos fascículos posteriores do nervo mediano, enquanto em 21 espécimes, dos fascículos póstero-laterais. Em 2 espécimes, estavam presentes dois ramos para o NIA. O NIP foi estudado em 30 membros, identificamos sua origem no nervo radial em todos os membros. Em 23 membros, os ramos para o músculo supinador originaram-se do NIP proximalmente à arcada de Fröhse, em 7 membros distalmente.

**Conclusão:**

O RPQ esteve presente de forma confiável em todos os antebraços dissecados e apresentou variações apenas em seu diâmetro. O RPQ pode ser transferido para NIP sem tensão em todos os espécimes, mesmo com amplitude total de movimento do antebraço. ***Nível de Evidência IV; Série de Casos.***

## INTRODUCTION

The anterior interosseous nerve (AIN) emerges from the posterior margin of the median nerve in variable sites. It initially runs parallel to the median nerve and distally follows a path between the flexor pollicis longus muscle (FPL) laterally and the flexor *digitorum profundus* (FDP) medially while innervating them. The AIN has a constant branch to the index finger FDP and partially innervates the long finger FDP. The ring and small finger FDP are innervated by the ulnar nerve. After the FDP and FPL branches, the AIN follows the anterior interosseous artery lying over the anterior face of the interosseous membrane and innervates the pronator quadratus muscle (PQ). The thinner terminal branch passes through the dorsal border of the PQ and has sensitive branches to the carpal joints.^1,2^ Considerable variations can be found regarding the proportion of FDP innervation by the median and ulnar nerves.^1,2^ Most AIN axons to the PQ are motor in its nature and could be used to reinnervate paralyzed muscles.^3^ Injuries to the radial nerve in the lower third of the arm or proximal forearm generally can be directly repaired or reconstructed with nerve grafts with good functional outcomes. Outcomes in surgical repair of the radial nerve are usually better than median and ulnar nerves due to its majority of motor fibers and not innervating intrinsic muscles of the hand.^4,5^ However, high radial nerve injuries, close to the axilla or posterior fascicle injury of the brachial plexus, are especially problematic. Because of the distance from target muscles and time necessary for reinnervation of extensor muscles in the forearm, these lesions usually generate functional impairments.^4–8^

Nerve injuries are managed by direct repair, nerve grafts, tendon transfers and free functioning muscle transfers. However, some nerve injuries are not amenable to primary repair and nerve grafts does not provide satisfactory results. This includes proximal nerve lesions, extended zone of injury with large gap between stumps, and idiopathic paralysis or neuritis with no healthy nerve fibers proximally.^2-4,6,7^ In brachial plexus injuries with extended gap between stumps, there may be not sufficient time for regenerating axons to reach the target muscle motor plate before they become permanently resistant to reinnervation. This prolonged period of denervation makes the target muscles susceptible to irreversible degeneration and fibrosis to the terminal motor plates.^4–6^

Tendon transfers were performed as the first line of treatment for radial nerve palsies but may be limited by often inconsistent results.^4, 6-9^ Plate et al^9^ considered advantages of nerve transfers over tendon transfers: use of expendable or redundant donor nerves over sacrificing a donor muscle; tendon transfers require extended dissection; and the difficulty of setting adequate length and tension in tendon transfers. Moreover, tendon transfers have been frequently associated with stiffness, biomechanical muscle imbalance, fibrosis, and vascular compromise. In radial nerve palsies, at least three separate donor tendons are needed for restoring wrist, fingers, and thumb extension. On the other side, nerve transfers require more time for target muscle reinnervation.^4–8^ Due to the proximity of median nerve to the radial nerve branches in the elbow it has been used for restoring radial nerve function.

Nerve transfers to restore wrist and fingers extension has been reported using donors such as median nerve branches to the pronator teres (PT), flexor carpi radialis (FCR), palmaris longus (PL), and flexor *digitorum superficalis* (FDS), AIN branches to the PQ; and radial nerve branches to the supinator.^3-5, 7-11^ Good functional outcomes have been reported from clinical series of nerve transfers to the posterior interosseous following high radial nerve palsies and brachial plexus injuries.^3-5, 7-11^ Despite encouraging clinical reports, few anatomical studies were conducted in this regard. The objective of this study was to analyze the characteristics and anatomical variations of the AIN innervation and evaluate the feasibility of transferring the PQ branch to restore fingers and wrist extension regarding tension in the repair and distance to target muscles.

## MATERIALS AND METHODS

Fifty upper limbs of 25 male adult cadavers were dissected, 20 were prepared by intra-arterial injection of 10% glycerin and formaldehyde solution and five were fresh cadavers. Each forearm was dissected with full elbow extension, wrist in neutral and forearm in pronation. No specimen had evidence of previous deformity, surgical procedures, or traumatic injuries in the studied area. Skin and fascia of the distal third of the arm, forearm and wrist was removed.

The median nerve was identified in the arm and dissected from proximal to distal. Biceps aponeurosis was sectioned, and the PT humeral head was detached distally and retracted. FCR and PL tendons were severed in their distal third to increase exposure of their motor branches. Median nerve branches to the PT, FCR, PL, FDS and AIN branches to the FDP, FPL and PQ were dissected after longitudinal division of the FDS and its fibrous arch. Anatomical variations of the AIN and its branches were recorded.

**Figure 1:**
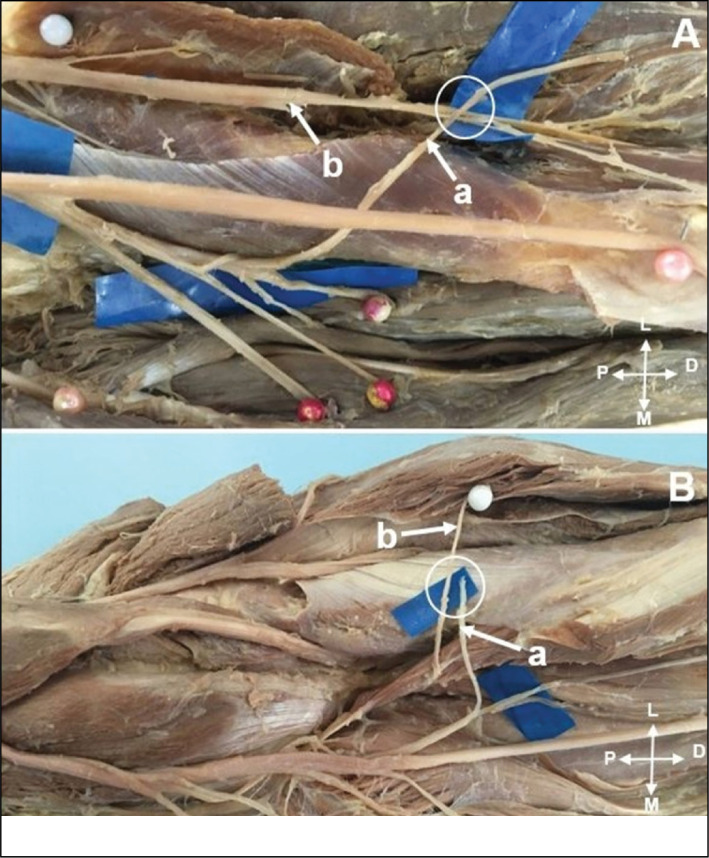
In vitro representation the feasibility of transferring the pronator quadratus branch (PQB) of the anterior interosseous nerve (AIN) (A); to the posterior interosseous nerve (PIN) (B).

Source: author.

**Figure 2:**
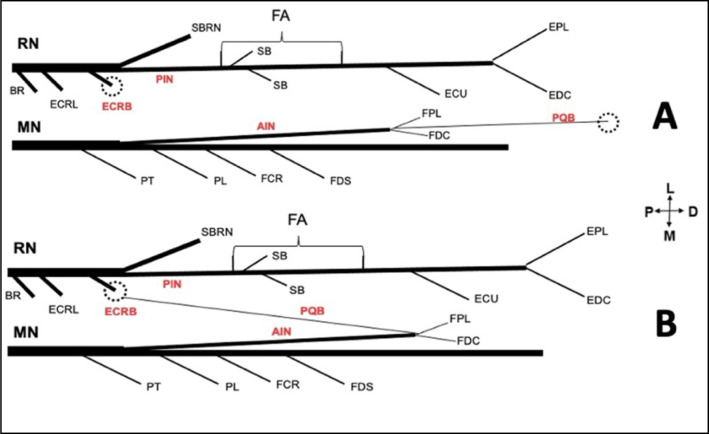
Schematic representation of the transfer of the PQB of the AIN to the PIN.

Source: author.

The radial nerve was identified in the arm between the brachialis and brachioradialis (BR) muscles. Motor branches to the brachialis, BR, extensor carpi radialis longus (ECRL), extensor carpi radialis brevis (ECRB), superficial branch of the radial nerve, posterior interosseous nerve (PIN) and its branches to the supinator were identified. Vascular structures were not preserved to facilitate nerve dissection. The following measures with ruler and digital pachymeter were performed: (1) forearm length measured from the center of a line between the medial and lateral epicondyles (intercondylar line) to the center of a line between the radial and ulnar styloid processes; (2) distance between the medial epicondyle and the site of AIN origin; (3) length of the PQ motor branch from its origin to the proximal margin of the PQ, and width 1cm after its origin. In vitro evaluation of in vivo feasibility of transferring the PQ branch of the AIN to the PIN (Figure 1) without tension was performed. (Figures 2A and 2B)

To facilitate the reader's understanding, the figures were identified with the letters and the following meanings: P = proximal; D = distal; M = medial; L = lateral.

The present study was approved by the Research Ethics Committee, CAAE 70128223.0.0000.5373.

## RESULTS

**Figure 3:**
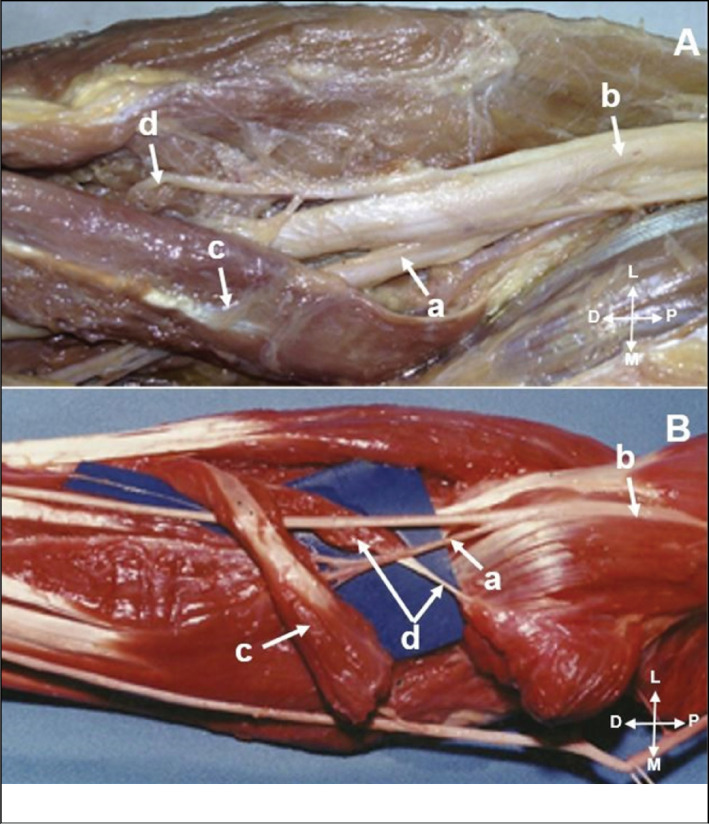
A) The anterior interosseous nerve (a); originated from the posterior fascicles of the median nerve (b); Humeral head pronator teres muscle (c). B) The anterior interosseous nerve (a); originated in the median nerve (b); proximal to the pronator teres muscle. Humeral head of the pronator teres muscle (c); ulnar head of the pronator teres muscle (d).

Source: author.

**Figure 4:**
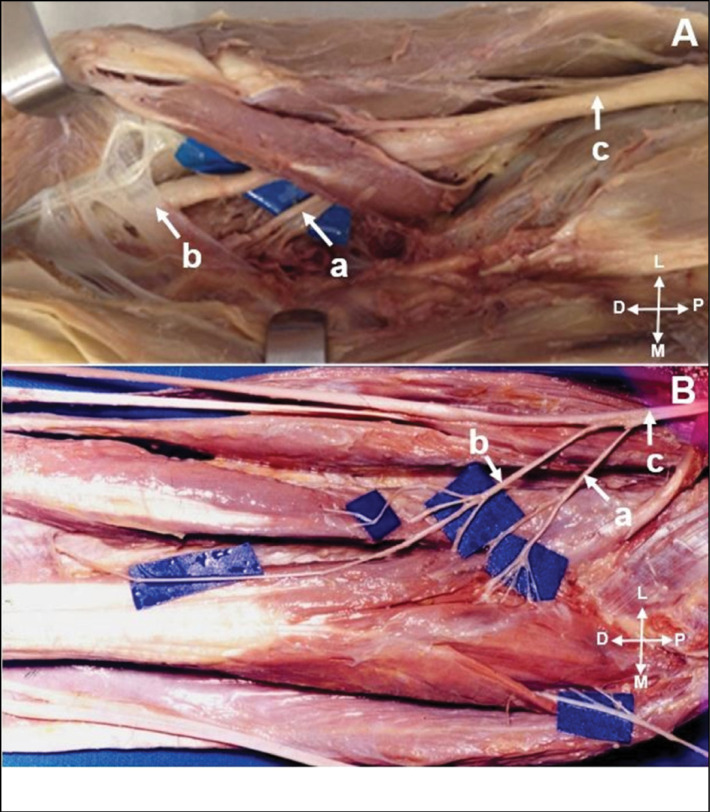
A) The anterior interosseous nerve (a); ramification proximal to the flexor digitorum superficialis arcade (b); Median nerve (c). B). In two members we found two anterior interosseous nerve branches (a and b); originated from median nerve (c).

Source: author.

**Figure 5:**
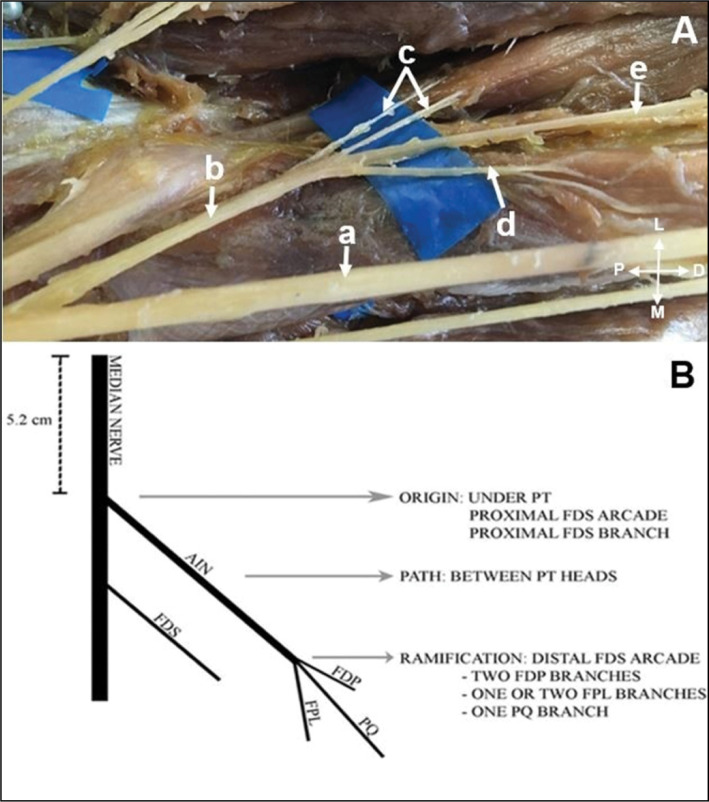
A) Median nerve (a); Anterior interosseous nerve (AIN) (b); branches to flexor digitorum profundus (FDP) (c); branch to Flexor pollicis longus (FPL) (d); branch to pronator quadratus muscle (PQ) (e). B) Schematic representation of most common anatomical pattern of anterior interosseous nerve origin, path, and ramification.

Source: author.

The median forearm length was 26.2 (± 2.7cm). The mean origin of the AIN from the median nerve was 5.2cm distal to the intercondylar line (ranging 1.5-7.5cm). In 29 members, the anterior interosseous nerve originated from the posterior fascicles of the median nerve (Figure 3A). In 21 members from the posterolateral fascicles. (Table 1) The AIN origin was proximal to the PT muscle in 12 forearms (Figure 3B) and distally in 6. In 32 limbs, it was located under the PT muscle belly (Figure 4A) (Table 1). The AIN ramification was proximal to the flexor digitorum superficialis arcade (Figure 4A) in 24 and distal in 26 forearms (Table 1). The anterior interosseous nerve was located between the humeral and ulnar heads of the pronator teres muscle in 41 forearms (Figure 3B) (Table 1). In two members we found two AIN originated from median nerve (Figure 4B). The AIN divides into branches for the FPL, FDC and branch to PQ muscle (Figures 5A and 5B). Regarding the number of branches to the FPL and FDP that penetrated different sites of the muscle bellies. The patterns of anterior interosseous nerve ramification were described in Table 2.

**Table 1:** Major anatomical data of anterior interosseous nerve.

Origin from median nerve	Distal to the ICL 5.2cm (1.5-7.5cm)			
From MN fascicles	Posterior 29	Postero-lateral 21		
Origin in relation to PT	Proximal 12	Distal 6	Under 32	
Position in relation to PT	Between humeral and ulnar heads 41	Posterior to both heads 2	Ulnar head absent	
			Posterior to humeral head 5	Through humeral head 2
Origin in relation to FDS arcade	Proximal 40			
Origin in relation to FDS branch	Proximal 38	At the same level 8	Distal 3	Between FDP and FPL 1
Ramification in relation to FDS arcade	Proximal 24	Distal 26		

ICL: intercondylar line; MN: median nerve; PT: pronator teres; FDS: flexor digitorum superficialis; FDP: flexor *digitorum profundus*; FPL: flexor pollicis longus.

**Table 2:** Patterns of anterior interosseous nerve ramifications.

Number of specimens	Branches for the FPL	Branches for the FDP
6	1	1
14	1	2
14	2	2
7	1	3
5	2	3
3	3	3
1	2	6

FDP - flexor *digitorum profundus.* FPL - flexor *pollicis longus*.

**Figure 6:**
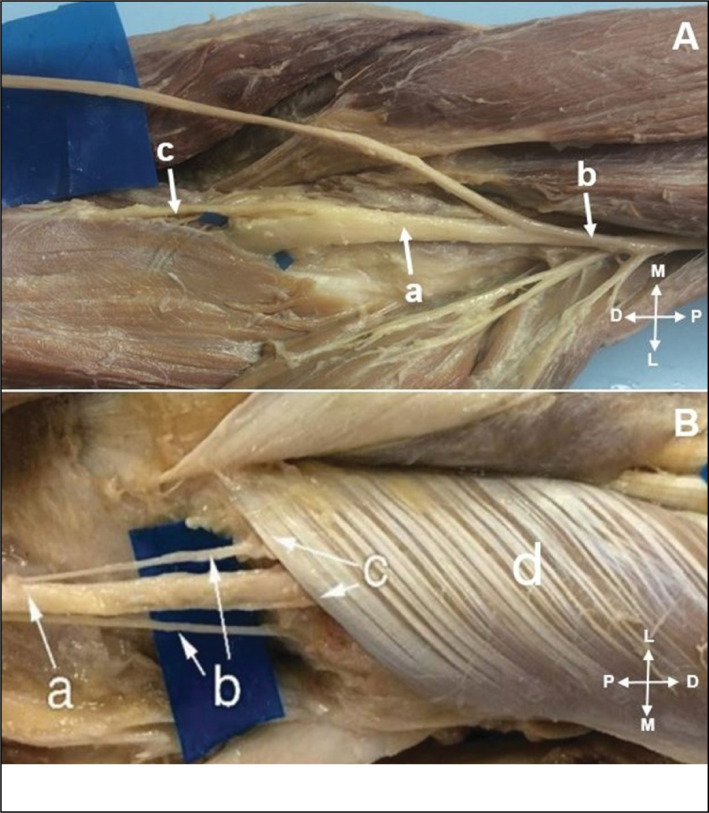
A) Posterior interosseous nerve (a); originated from radial nerve (b); branch for extensor carpi radialis brevis (c). B) Branches for supinator muscle (b); originated from posterior interosseous nerve (a); Fröhse arcade (c); supinator muscle (d).

Source: author.

The PIN was studied in 30 limbs, we identified its origin in the radial nerve in all limbs (Figure 6A). In 23 limbs the branches to the supinator muscle originated from the PIN, proximal to the Fröhse arcade (Figure 6B). In 7 members distal the arcade. We identified the origin of the extensor carpi radialis brevis in the PIN in 16 of 30 limbs, in the superficial branch of the radial nerve in 10, and in the radial nerve in only 4.

In 14 specimens, the AIN branch to the PQ was rerouted to the PIN to evaluate the possibility of nerve transfer without tension. The transfer was assessed during forearm and elbow range of motion. The anatomical measurements of length and diameter of donor and receptor nerves was described in Table 3.

**Table 3:** Anatomical measurements of length and diameter of donor and receptor nerves.

	Forearms	Numbers of branches	Average diameter (in mm)	Average length (in mm)
AIN	50	1 (48 limbs) 2 (2 limbs)	1.7 ± 0.5 (14 limbs)	3.5 ± 2.7
PQ branch	50	1 (50 limbs)	1.0± 0.5 (14 limbs)	10.8 ± 4.5
PIN	30	1 (30 limbs)	3.0 ± 0.5 (30 limbs)	5.2 ± 2.8

AIN – Anterior interosseous nerve. PIN – Posterior interosseous nerve. PQ – Pronator quadratus. The AIN was measured from its origin from the median nerve to the last ramification. The PQ branch was measured from the AIN ramification to the proximal margin of the PQ.

## DISCUSSION

Cadaveric studies reveal potential donor and receptor nerves for transfers to restore wrist and fingers extension. Several parameters are significant and can be evaluated during dissections, such as donor and receptor nerves length, diameter, and possibility to mobilize; axon count; and synergic relations.^3-7, 10,11^ Ustum et al^3^ evaluated 10 limbs of 5 cadavers and analyzed the feasibility of median nerve transfers to restore unrepairable radial nerve and brachial plexus injuries. The authors considered the PQ branch the most adequate for transfers due to its length and the FDP branches are too short for neurotizations. It could be easily transferred to the PIN proximal to the ECRB branch, to the ECRB branch, or to the PIN distal to the supinator branch. However, its diameter is approximately half the size of the PIN and could be applied only to reinnervate part of the PIN before or after the extensor *carpi ulnaris* (ECU) branch. These authors suggest that the number of donor axons can be improved by associating the FPL branch, the PT branch, and the PQ branch.

In our study, the PQ branch was also the longest donor and was always present. It could be transferred to the extensor digitorum communis and extensor pollicis longus branches in a straightforward manner, proximal or distal to the ECU branch. The authors recommend the transfer distal to the ECU and supinator branches to provide adequate donor axons targeting fingers and thumb extension. Transfer without tension is also possible to the ECRB branch, which has adequate length and similar diameter. Bertelli et al^12^ performed PQ branch transfers to the ECRB branch to restore wrist extension in 28 patients with C5-8 brachial plexus injuries.

We have identified more than one FPL branch in 23 and more than one FDP branch in 44 forearms. The average FPL branch measured 1.2cm longer than the FDP branches. Simulating the neurotization procedure in 14 specimens, the authors sectioned the PIN branch next to its origin and transferred without tension the FPL and FDP branches from the site of innervation of target muscles.

We identified a mean PQ branch diameter of 1.0mm ± 0.5 and it was approximately 33.5% of the mean PIN diameter of 3.0mm ± 0.5. We identified a mean PQ branch diameter of 1.0mm ± 0.5 and it was approximately 33.5% of the mean PIN diameter of 3.0mm ± 0.5. Nevertheless, several authors reported good outcome of nerve transfers even with mismatch between fiber diameters. As an example, the triceps branch of the radial nerve transfer to the axillary nerve has reported good outcomes even with a 50% smaller diameter of the donor nerve.^13, 14^ Narakas and Hentz^15^ reported a 15-30% loss in nerve fibers after a nerve transfer without graft and this could further impact the availability of donor axons. Despite, De Medinaceli^16^ stated that only 20-30% of muscle fiber is compatible with normal muscular strength. Furthermore, Jian et al^17^ described that axons in the proximal stump can multiply and increase up to 3-4 times its number. In a study with rabbits, Lutz et al^18^ demonstrated that axonal multiplication between donor and receptor nerves was 1:3 ratio. Also, Tötösy de Zepetnek et al^19^ observed that at least 30% of original motor neurons are necessary for normal muscle strength in rats. Therefore, donor nerve should have at least 30% the diameter of the receptor nerve. Even with fiber mismatch between the PIN and PQ branch, muscle strength that needs to be recovered for fingers and thumb extension is small since tension for release functions of the hand is minimal.

## CONCLUSION

The PQ branch of the AIN was reliably present in all dissected forearms and presented variations only in its diameter. The PQ branch could be transferred to the PIN without tension in all specimens even with full range of motion of the forearm.
